# Control of DNA Replication Initiation by Ubiquitin

**DOI:** 10.3390/cells7100146

**Published:** 2018-09-20

**Authors:** Esperanza Hernández-Carralero, Elisa Cabrera, Ignacio Alonso-de Vega, Santiago Hernández-Pérez, Veronique A. J. Smits, Raimundo Freire

**Affiliations:** 1 Unidad de Investigación, Hospital Universitario de Canarias, Instituto de Tecnologías Biomédicas, Ofra s/n, 38320 La Laguna, Tenerife, Spain; esperanza.carralero@gmail.com (E.H.-C.); ecabrera@ull.edu.es (E.C.); igna.adv@gmail.com (I.A.-d.V.); santibio@gmail.com (S.H.-P.); vsmits@ull.edu.es (V.A.J.S.); 2 Division of Oncogenomics, The Netherlands Cancer Institute, Plesmanlaan 121, 1066 CX Amsterdam, The Netherlands

**Keywords:** ubiquitin, DNA replication Initiation, ubiquitin ligases, ubiquitin hydrolases, proteasome

## Abstract

Eukaryotic cells divide by accomplishing a program of events in which the replication of the genome is a fundamental part. To ensure all cells have an accurate copy of the genome, DNA replication occurs only once per cell cycle and is controlled by numerous pathways. A key step in this process is the initiation of DNA replication in which certain regions of DNA are marked as competent to replicate. Moreover, initiation of DNA replication needs to be coordinated with other cell cycle processes. At the molecular level, initiation of DNA replication relies, among other mechanisms, upon post-translational modifications, including the conjugation and hydrolysis of ubiquitin. An example is the precise control of the levels of the DNA replication initiation protein Cdt1 and its inhibitor Geminin by ubiquitin-mediated proteasomal degradation. This control ensures that DNA replication occurs with the right timing during the cell cycle, thereby avoiding re-replication events. Here, we review the events that involve ubiquitin signalling during DNA replication initiation, and how they are linked to human disease.

## 1. Introduction

During DNA replication, two identical copies of all double-stranded DNA molecules are produced. This process is critically linked to cell division, as the duplicated DNA needs to be equally divided over the two daughter cells that must obtain the same genetic information as the parent cell. Errors during DNA replication, including under- and over-replication events, may result in genomic alterations. To avoid such situations, initiation of DNA replication is carefully regulated at the so-called DNA replication origins, regions in the genome where replication starts. Under-replication is prevented by the existence of many origins that can be potentially activated, and over-replication is avoided by restricting origin activation to only once per cell cycle [1]. Therefore, DNA replication control, and more specifically DNA replication initiation, is a very controlled cellular process, crucial for maintaining the genomic integrity of dividing cells. This exquisite control of replication initiation depends on many factors and modifications, among which ubiquitination plays a key role.

## 2. Ubiquitin Modification

Ubiquitination is a reversible process which generates an isopeptide link between the C-terminal glycine of the ubiquitin molecule, a highly conserved protein that exists in all eukaryotic cells, and the ε-amino group of a substrate lysine residue [2]. The ubiquitin molecule itself contains seven lysine residues (Lys6, Lys11, Lys27, Lys29, Lys33, Lys48, and Lys63) onto which additional ubiquitin molecules can be elongated to chains with diverse lengths and by different linkages [3]. Different types of modifications by ubiquitin exist: single or multiple monoubiquitination, diubiquitination, and polyubiquitination. While mono- and diubiquitination always lead to functional changes in the modified proteins, such as (in)activation or a change in the subcellular location, the effect of polyubiquitination can either change the function of the protein or target the modified protein for degradation, depending on which ubiquitin lysine is used for the elongation. One of the most studied ubiquitin chains is the elongation with Lys48 conjugation that sends the proteins to the 26S proteasome, a protein complex that degrades unnecessary or damaged proteins by proteolysis. In contrast, ubiquitin chains conjugated via Lys63 have not been not associated with degradation, but rather with protein functional changes. The function and fate of other ubiquitin linkages is not yet clear but Lys29 and Lys11-linked chains were found to be related to protein degradation [4].

Ubiquitination is catalysed by the sequential action of three enzymes: E1 or activating enzyme, E2 or conjugating enzyme, and E3 or ubiquitin ligase, involved in the conjugation of ubiquitin to the target protein [2]. Depending on the E3, the ubiquitin ligase can bind both the E2 enzyme and the target protein after which the E2 transfers the ubiquitin to the substrate or, the E3 itself has a dual role in substrate recognition and ubiquitin conjugation. The human genome encodes two E1s, fewer than 60 E2s and more than 600 different E3s, in which the E3s determine the specificity for the different substrates [5,6].

The most important E3 ubiquitin ligase complexes regulating cell cycle progression in mammalian cells (by catalysing most known cell cycle ubiquitination events) are the anaphase promoting complex/cyclosome (APC/C) and the Cullin-RING E3 Ligase (CRL) [7,8]. The APC/C is a multi-subunit E3 ubiquitin ligase that polyubiquitinates its substrates with Lys48- and Lys11-linked ubiquitin chains. APC/C substrates contain specific recognition motifs (D-boxes and KEN boxes) and are targeted for degradation by the proteasome. Vertebrate APC/C is composed of at least 14 different protein core subunits, and is only fully active as a ubiquitin ligase with one of the cofactors Cdc20 or Cdh1. Cdc20 is the activator of APC/C during early mitosis and is thereby a key component in allowing mitotic exit [9]. At the end of the mitosis, the mitotic phosphatase Cdc14 is able to dephosphorylate and activate Cdh1, which then competes with Cdc20 for binding to APC/C. APC/C-Cdh1 subsequently drives cells out of mitosis, playing a key role in G1 phase [10].

CRLs use one of the six different Cullins existing in humans as a scaffolding subunit to bring the E2 enzyme and the substrate together. The SCF (or CRL1) complex is composed of four subunits: Cullin 1, Roc1, Skp1, and one of many F-box protein substrate adaptors that confer substrate specificity (e.g., Skp2 or βTrCP). In contrast to APC/C, the SCF-Skp2 complex starts functioning at the G1/S transition and stays active throughout S phase [7,8].

Conversely, ubiquitin hydrolases or deubiquitinating enzymes (DUBs) antagonize the ubiquitin conjugation carried out by the E3 ubiquitin ligases. The human genome encodes nearly 100 DUBs grouped into five families: Otubain domain-containing proteases (OTU), Ubiquitin-specific proteases (USPs), Ubiquitin C-terminal hydrolases (UCHs), Machado–Joseph domain-containing proteins (Josephin), and JAB1/MPN/Mov34 proteases (JAMM). The first four families are cysteine proteases, whereas the last one consists of metalloproteases [11]. While several DUBs controlling different aspects of cell cycle have been described, to date USP7/HAUSP is one of the most important regulators during DNA replication [12]. Initially, USP7 was described as an ubiquitin hydrolase for the tumour suppressor protein p53, whereas in later studies USP7 was shown to have a much higher affinity for MDM2/HDM2 [13,14]. From then on, a variety of additional USP7 substrates have been reported. Recently USP7 was shown to recognize and subsequently deubiquitinate proteins that were previously modified by Sumo, a ubiquitin-like molecule [15].

## 3. Initiation of DNA Replication

Eukaryotes contain multiple DNA replication origins grouped in so-called replicons. Replication begins with the process called origin licencing, a mechanism in which a group of proteins binds to an origin in an ordered manner allowing the start of DNA replication [16]. Notably, only a subset of all licensed origins gets activated and fired in each S phase of the cell cycle. This decision of which origins are fired differs between cell types, and even varies within the same cell population, thereby allowing a possible adaptation to environmental signals and different types of cell stress [17,18].

**Figure 1:**
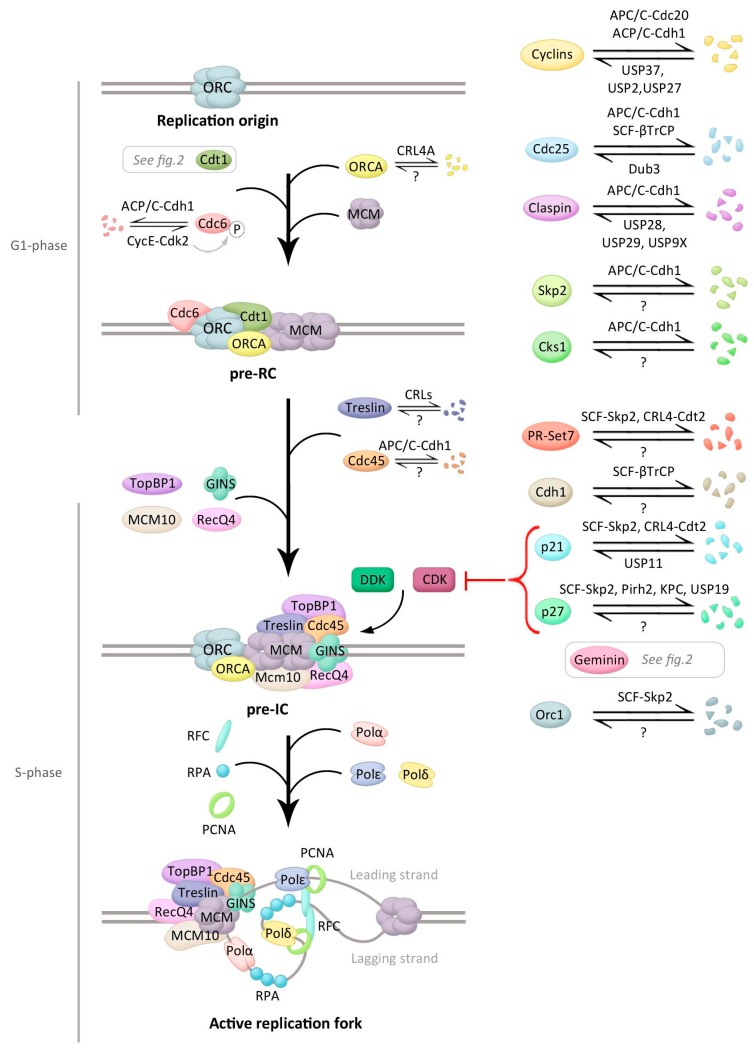
Main events during initiation of DNA replication and control by ubiquitination. Schematic presentation of the events occurring during DNA replication initiation (early events at the top and later at the bottom) with the main regulators in each stage. Proteins involved in this process that are modified by ubiquitin, and if known, the ubiquitin ligase(s) and/or the deubiquitinating enzyme(s) (DUB(s)) controlling this modification, are depicted. See text for details.

Licensing of DNA replication origins starts in late mitosis and the early G1 phase, when the origin recognition complex (ORC), formed by six subunits (Orc1–6), recognizes and binds to replication origins (Figure 1), a process that is stabilized by ORCA/LRWD1 [19,20]. The assembly of ORC complexes onto the replication origins allows the recruitment Cdt1 and the AAA+ ATPase Cdc6, which enhances the loading of the MCM (composed of Mcm2–7 subunits) helicase complex onto origins as an inactive head-to-head double hexamer, in an ATP hydrolysis-dependent reaction [19]. Upon full assembly of this so called pre-replicative complex (pre-RC), DNA replication origins are licensed and capable to replicate (Figure 1). The efficiency of licensing is controlled by additional regulation of chromatin structure near DNA replication origins. Among the different histone-modifying enzymes controlling this process, the methyltransferase PR-Set7/Set8 has emerged as a critical player in origin licensing. PR-Set 7 monomethylates histone H4 at lysine 20 (H4K20me1) and promotes ORC, Cdc6, and MCM association to the origins. H4K20me1 modification can serve as a template for di- and tri-methylation by both Suv4-20h1 and 2 methyltransferases, and H4K20me3 was suggested to promote ORCA binding, hence stimulating origin licensing [21].

Activation of the pre-RC includes the binding of the DNA polymerase machinery to the complex and the dissociation of the double Mcm2–7 hexamer into two active MCM hexamers that then form two replisomes able to unwind DNA and start two replication forks in each replication origin [18]. This step requires the activity of the S phase kinases CDK and DDK, also known as the Cdc7-Dbf4 complex, in which Dbf4 and Cdc7 are regulatory and catalytic subunits, respectively. Both CDK and DDK phosphorylate the MCM complex and this phosphorylation triggers recruitment of other proteins forming the pre-initiation complex (pre-IC) that starts DNA replication [22]. Among these proteins, Cdc45 and the GINS complex associate to the MCM complex, thereby forming the CMG (Cdc45/MCM/GINS) complex, the functional helicase [18]. CDK or DDK additionally regulate other pre-IC proteins. Cdc7 for example interacts with and phosphorylates Claspin, an essential mediator protein in the DNA replication checkpoint, responsible for the activation of Chk1 [23]. Claspin also recruits Cdc7 for efficient initiation of DNA replication in unperturbed cells [24]. By phosphorylating Treslin, CDKs promote binding of Treslin to DNA topoisomerase 2-binding protein 1 (TopBP1), required for the activation of the CMG helicase. Both Treslin and TopBP1 are components of the pre-IC required for the activation of the MCM hexamer. CDKs additionally activate ATP-dependent DNA helicase Q4 (RecQ4, also called RecQL4) by phosphorylation. RecQ4 is, together with Mcm10, required for the formation and activation of the CMG complex [25].

The active CMG complex unwinds double-stranded DNA to initiate DNA synthesis and additionally triggers the recruitment of several proteins, such as DNA polymerases, replication factor C (RFC), proliferating cell nuclear antigen (PCNA), and replication protein A (RPA) [18]. Mcm10 is also required for the loading of RPA and DNA polymerases Polα (involved in DNA initiation) and Polδ (involved in recessive DNA replication) [25,26]. The two DNA strands are synthesized by different mechanisms as the leading strand is continuously replicated, whereas the lagging strand is replicated in a discontinuous way. The primase enzyme generates the initial RNA primer, followed by a short stretch of DNA synthesized by Polα. RFC then binds to the primer template junction and catalyses the loading of replication factor PCNA, which encircles DNA [27], thereby recruiting the replicative polymerases Polδ or Polε and enhancing its processivity [28].

## 4. Ubiquitination Control during Initiation of DNA Replication

As mentioned, initiation of DNA replication is a strictly regulated process in which control by ubiquitination plays an important role (Figure 1). Protein levels of Orc1, the largest subunit of the human ORC complex and important for recognition of replication origins, oscillate during the cell cycle of proliferating cells, partly by ubiquitin control. Orc1 is expressed in an E2F-dependent manner and recruited to chromatin as cells exit mitosis and replication origins start being recognized [29,30]. During the G1/S transition, Orc1 is ubiquitinated by the SCF-Skp2 complex and targeted for degradation, which avoids the recognition of new DNA replication origins [31]. Upon the exit from mitosis, Orc1 is deubiquitinated and bound to chromatin [32]. After ORC recognition, the ability of Cdc6 to recruit MCM to replication origins is controlled by both transcriptional regulation and CDK-regulated subcellular localization of Cdc6 [33,34]. Moreover, ubiquitin-mediated degradation of Cdc6 by the APC/C-Cdh1 early in G1 prevents its accumulation until late G1 [35]. APC/C-Cdh1 additionally targets Geminin, the inhibitor of Cdt1, for proteolysis, helping Cdt1 to recruit MCM complexes to the origins as will be discussed below [18]. APC/C-Cdh1 additionally maintains the CDK activity low, required for pre-RC assembly, through different mechanisms. First, APC/C-Cdh1 targets the CDK activator Cdc25A for degradation by ubiquitination [36]. Second, APC/C-Cdh1 mediates the accumulation of the CDK inhibitors (CKIs) p21 and p27 by promoting the degradation of the cofactor Cks1 and the Skp2 subunit of the SCF ubiquitin ligase complex responsible for ubiquitination and subsequent degradation of these CKIs (Figure 1) [37].

Upon entry into S phase, APC/C-Cdh1 becomes inactive and no longer targets Cdc6 for proteasomal degradation. Moreover, Cdc6 becomes phosphorylated at Ser54 by cyclin E-Cdk2, which disrupts the interaction between Cdc6 and Cdh1 and furthers protects Cdc6 from ubiquitin-mediated degradation [35]. Paradoxically, Cdc6 levels are higher in the S and G2 phases as compared to G1, when licencing occurs. However, during the S phase different mechanisms target Cdc6 for cytoplasmic import, where it is unable to participate in origin licensing [38]. Moreover, during G2 and mitosis the E3 ligase complex SCF-cyclin F prevents DNA re-replication by interaction with and promoting ubiquitin-mediated proteasomal degradation of Cdc6 [39].

The inactivation of APC/C-Cdh1 prevents the degradation of Geminin as will be discussed below. Subsequent Geminin accumulation disrupts the interaction between ORCA and Cdt1 at the origins and additionally, ORCA becomes polyubiquitinated by Cul4A-DDB1 and is sent for degradation during the S phase. ORCA ubiquitination occurs at its WD40 repeat, a domain required for ORCA chromatin association and Orc2 binding. Moreover, Orc2 promotes cellular ORCA stabilization by interacting only with non-ubiquitinated ORCA, thereby avoiding association with E3 ligases like Cul4A-DDB1 [40]. Furthermore, the ubiquitin hydrolase Dub3 counteracts both APC/C-Cdh1 and SCF-βTrCP-dependent degradation of Cdc25A by deubiquitinating and thus stabilizing this phosphatase [41].

The kinase DDK is also controlled by ubiquitination. In budding yeast, Cdc7 levels are constant during the cell cycle but Dbf4 protein levels oscillate. Dbf4 levels are regulated by proteasomal destruction by APC/C-Cdc20 and Dbf4 is thereby absent during G1, peaks in G1/S and remains high through late mitosis [42,43,44,45]. Dbf4 was also reported to be stabilized after stalled DNA replication, due to degradation of Cdh1 [46]. The Cdc7 regulator Claspin is stabilized during S phase and degraded in mitosis. Claspin levels are consequently low in early G1 cells [47,48,49,50]. During G1, APC/C-Cdh1 is responsible for the ubiquitin-mediated degradation of Claspin, while ubiquitin hydrolase USP28 specifically counteracts this ubiquitination [51]. Also DUBs USP29 and USP9X function to stabilize Claspin and lack of the DUBs consequently leads to a failure in S phase progression/DNA replication [52,53].

Further regulations during the initiation of DNA replication involve Treslin and RecQ4. Treslin phosphorylation by CDK during S phase promotes its stabilization as the nonphosphorylated form is degraded by the proteasome in an Ensa-dependent manner [54]. Ensa is a component of the Greatwall/Ensa/PP2A-B55 pathway, in which Greatwall kinase activates Ensa by phosphorylation in mitosis. Upon activation, Ensa inhibits PP2A-B55δ phosphatase, and controls the binding of substrates to the CRLs [55]. RecQ4 protein levels are also cell cycle regulated. RecQL4 Cdk1/2-dependent phosphorylation in S/G2 phase stimulates ubiquitination by CRL4, which enhances the recruitment of RecQ4 to double strand breaks and regulates repair pathway choice [56]

Additionally, a redundant mechanism avoids origin licensing throughout S phase as global levels of PR-Set7, and therefore H4K20me1, decrease at G1/S, remaining low during the S and G2 phases as result of ubiquitin-mediated proteolysis by both CRL4-Cdt2 and SCF-Skp2 [57,58]. Moreover, in yeast there is evidence of non-proteolytic Mcm10 ubiquitination in lagging-strand synthesis, through modulation of protein–protein interactions. Specifically, the monoubiquitination of Mcm10 at two distinct lysines facilitates the recruitment of the elongation polymerases ε and δ via an increased Mcm10-PCNA affinity, while the affinity of Mcm10 for the primase Polα diminishes. The latter is an example of modification by ubiquitin regulating DNA replication by changing interactions rather than sending proteins for degradation by the proteasome [59].

Ubiquitination also is important at the G1/S transition, regulating CDK activity by controlling the protein levels of cyclins and CKIs. SCF, together with different F-box proteins, promotes the degradation of cyclin D1 [60]. Phosphorylation of cyclin D1 at Thr286 by GSK-3β determines its nuclear export and subsequent ubiquitination and proteolysis [61,62]. In contrast, USP2 promotes cyclin D1 stabilization by antagonizing the ubiquitination [63]. Likewise, cyclin E abundance is controlled by ubiquitination, involving different ubiquitin ligases that target cyclin E for proteasomal degradation: a Cullin 3-dependent ligase and SCF-Fbxw7 [64,65]. To prevent this degradation, USP27 interacts with and deubiquitinates cyclin E [66]. Moreover, USP37 contributes to the stabilization of cyclin A by counteracting APC/Cdh1 functioning. USP37 activity is stimulated by Cdk2-mediated phosphorylation at Ser628 in a positive feedback loop [67]. In addition to the SCF-Skp2 E3 ligase degrading p27 and p21 at the G1/S transition that was described earlier in this review, the E3 ubiquitin ligases Pirh2 and the KPC complex contribute to proteasomal degradation of p27 by ubiquitinating this CKI at the G1/S boundary [68,69]. USP19 in turn also contributes to p27 degradation by stabilizing KPC1 [70].

A number of processes control APC/C Cdh1 activity, several of which concerning ubiquitination. First, Cdh1 is phosphorylated by Plk1 and by Cdk2-cyclin A, thereby triggering its polyubiquitination by the SCF-βTrCP1 E3 ligase complex [71,72]. Moreover, APC/C-Cdh1 was demonstrated to autoubiquitinate using the E2 UbcH10 [73]. Finally, controlling Emi1 levels was shown to be important for the rapid inactivation of APC/C-Cdh1. Emi1 is a substrate but also an inhibitor of APC/C-Cdh1, and while Emi1 levels are low and the APC/C-Cdh1 activity is high during G1, at the G1/S transition Emi1 transcription rises in an E2F-dependent manner, resulting in high Emi1 levels and low APC/C-Cdh1 activity during the S and G2 phases [74,75]. At prophase, Emi1 is subsequently degraded by SCF-βTrCP [76,77].

Finally, while not the focus of this review, it is important to mention that ubiquitin modifications additionally have a key function in several aspects of DNA replication other than initiation. For example, ubiquitination of H2A by the ubiquitin ligase RNF168 is required for efficient replication fork progression during the unperturbed S phase [78]. Additionally, studies in *Xenopus* and yeast have shown that during DNA replication termination, disassembly of the replisome involves polyubiquitination of Mcm7 and chromatin removal by p97/VCP, a factor involved in the displacement of polyubiquitinated proteins from chromatin and the subsequent degradation of these proteins by the proteasome [79,80,81].

## 5. Cdt1 and Geminin Control to Avoid Re-Replication

**Figure 2:**
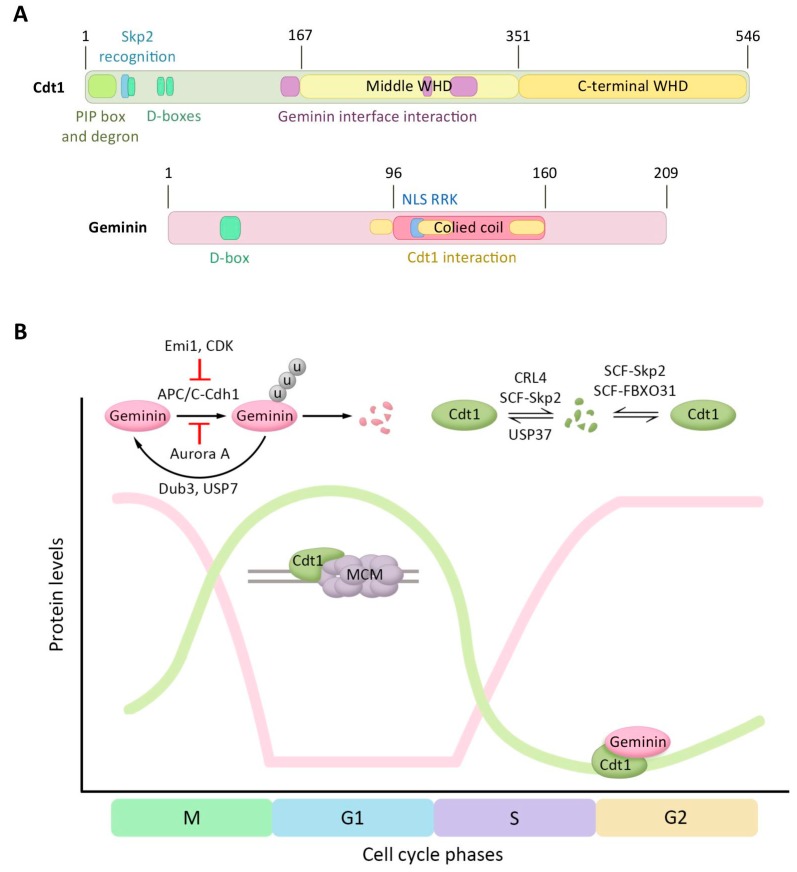
Regulation of Cdt1 and Geminin protein levels by ubiquitination-mediated degradation. (**A**) Domain structure of human Cdt1 and human Geminin. For Cdt1, the regulation motifs of the N-terminal domain (PIP box, Cdt2-recognizing degron motifs, the Skp2 recognition site, and the three destruction boxes), the middle winged helix domain (WHD), the C-terminal WHD, and the region interacting with Geminin are represented. Geminin structure contains a destruction box, a coiled-coil domain, a Cdt1 interacting region, and a nuclear localization signal (NLS). (**B**) Diagram presenting Cdt1 and Geminin protein levels, and its regulators, during the cell cycle. The y-axis presents the levels of the two proteins and the x-axis the progression through the cell cycle. The ubiquitin ligases and DUBs controlling these proteins, the recruitment of MCM by Cdt1, and the Geminin–Cdt1 interaction are additionally depicted.

Cdt1 and Geminin are two crucial players in the control of DNA replication licensing. The levels of both proteins are heavily controlled by ubiquitin-dependent proteasomal degradation, in this manner allowing the cells to prevent re-replication and deregulation in origin licensing. The Cdt1 orthologue in *Schizosaccharomyces pombe* was first identified by searching for novel target genes of the Cdc10 transcription factor [82]. Structurally Cdt1 is formed by three domains (Figure 2A), an N-terminal domain (residues 1–166) and two winged helix domains (WHD), one of them in the middle of the protein (residues 167–351) and the other in the C-terminal region (residues 352–546) [83,84]. The N-terminal domain mediates the interaction with other proteins. To date, four different E3 ligases are known to recognize the Cdt1 N-terminal domain and to promote its proteasome-dependent degradation during the S and G2 phases (Figure 2B). First, cyclin A-Cdk2 recognizes the cyclin-binding sequence/Cy-motif ArgArgLeu (aminoacids 68–70) and phosphorylates Cdt1 at Thr29 [85,86]. This phosphorylation allows the recognition of the SPARPALR site by Skp2 of the SCF-Skp2 complex that functions as a E3 ligase and triggers Cdt1 ubiquitination and proteasomal degradation during G2 phase [87,88]. Secondly, Cdt1 can be targeted for proteolysis in a PCNA-dependent manner by the E3 ligase Cul4-DDB1-Cdt2 (CRL4-Cdt2). PCNA binds to a consensus PCNA-interaction motif (PIP) located within the first 28 amino acids of Cdt1, which allows the recognition of the adjacent degron motif in Cdt1 by Cdt2 [88,89,90]. Once PCNA is loaded onto the DNA, in the S phase or after UV-irradiation, CRL4-Cdt2 mediates Cdt1 ubiquitination and degradation, avoiding relicensing of replication origins. Third, Cdt1 is ubiquitinated at its N-terminus by the E3 ligase SCF-Fbxo31 during G2 phase [91]. Ultimately, the three D-boxes present in Cdt1 are recognized by APC-Cdh1, mediating Cdt1 proteolysis when cells enter G0 [92]. A recent study reported the involvement of the ubiquitin hydrolase USP37 in controlling Cdt1 levels. USP37 downregulation destabilizes Cdt1 during G1 phase and the G1/S transition [93].

The degradation of Cdt1 additionally involves extraction of chromatin-bound Cdt1 in a manner dependent on p97/VCP [94]. Whereas Cdt1 phosphorylation promotes its degradation, the Cdt1 N-terminus is also acetylated by the acetyltransferases KAT2B and KAT3B during G1 phase, thus preventing Cdt1 ubiquitination and proteasomal degradation. Reversion of Cdt1 acetylation is mediated by the deacetylase HDAC11 [95]. The binding of Cdc7 to the Cdt1 N-terminal domain stabilizes Cdt1 on chromatin during early S phase. The interaction between Cdt1 and Cdc7 additionally leads to recruitment of Cdc7 to chromatin and the loading of Cdc45, providing a positive feedback loop. In the late S phase however, replication origin re-firing is prevented by promoting Cdt1 dissociation from chromatin upon Cdc7 kinase activity [96]. Although the binding site for Geminin is located in the middle domain of Cdt1, this binding site is not located at the WHD fold [83,84]. WHD domains are present in other pre-RC proteins/licensing factors such Orc1, Orc2, Cdc6, and Mcm6 [84]. Cdt1 WHDs consist of four α-helices and two β-strands that are mediating MCM complex recruitment at the DNA replication origins through the interaction with the C-terminal WHD domain of Mcm6 [97].

Geminin was originally described in *Xenopus* egg extracts as an inhibitor of DNA replication initiation that is destabilized in mitosis [98]. Geminin inhibits the replicative functions of Cdt1 from S phase until early mitosis by direct interaction [99]. This inhibitory licensing complex was suggested to be formed on the chromatin [100]. Geminin and Cdt1 both regulate MCMs loading at the origins of replication and replication licensing by controlling histone acetylation. Geminin inhibits licensing by histone acetylation suppression via Cdt1 in two different ways. During G1, Cdt1 recruits histone acetylase HBO1, which acetylates histone H4 and thereby induces chromatin decondensation and loading of MCM complex, a process reported to be repressed by Geminin. Moreover, the interaction of Geminin with Cdt1 promotes histone deacetylation by HDAC11 in the S phase [95,101].

Geminin is organized in three different regions (Figure 2A): a N-terminal region (residues 1–95), a central coiled coil domain (residues 96–160) and a C-terminal domain with uncharacterized function (residues 160–209) [83,102,103,104]. The N-terminus of Geminin contains a D-box (residues 23–31) that is recognized by the APC/C complex and conjugates ubiquitin to nearby lysine residues, leading to Geminin degradation at the exit of mitosis and during the G1 phase [98,105,106]. Geminin contains a nuclear localization sequence (NLS) and localizes in the nucleus. Whereas non mammalian Geminin contains an NLS located within the N-terminal domain, the mammalian Geminin NLS is located in the coiled-coil domain in an Arg–Arg–Lys sequence (RRK, residues 106–108) [104,107]. Importantly, Arg106 and Arg107 are important in the interaction of Geminin with Cdt1 and Cdt1 binding could therefore interfere with the nuclear localization of Geminin [107,108]. Although coiled-coil domains are simple structures that include two or more α-helical peptides, they are functionally highly versatile, and important for protein oligomerization. The Geminin homodimer is more unstable than other coiled-coil interactions, but was suggested be more stable upon interaction with Cdt1. In addition, the instability of the Geminin homodimer allows Geminin to heterodimerize with other partners providing additional mechanisms for Geminin regulation [109]. Indeed, Cdt1-Geminin heterotrimers have been reported [83,109].

Geminin protein levels are also regulated during the cell cycle (Figure 2B). At the metaphase/anaphase transition, Geminin is ubiquitinated and sent for degradation by the E3 ligase APC/C-Cdh1 that is active from the end of mitosis [98,110]. However, in earlier steps of mitosis, Geminin degradation is protected by two mechanisms: APC/C-Cdh1 inhibitor Emi1 blocks APC/C-Cdh1 activity, and Aurora A kinase phosphorylates Geminin at Thr25 to block its interaction with the APC/C-Cdh1 complex [111]. Recently, the ubiquitin hydrolases Dub3 and USP7 were identified to regulate the stability of Geminin. Depletion of Dub3 results in an increased number of re-replicating cells, demonstrating the importance of controlling levels of Geminin in avoiding re-replication and genomic instability [112].

The regulation of Cdt1 is crucial in preventing DNA re-replication and is carried out at two levels: protein stability and activity control. As described, Cdt1 levels oscillate during the cell cycle and are high in late mitosis and G1 phases, as is required for the formation of the pre-RC (Figure 2B). However, when the cells enter into the S phase, Cdt1 is targeted for proteasomal degradation to inhibit the activation of excess origins. After the S phase, Cdt1 starts to be stabilized in order to be ready for the next licensing cycle. Importantly, a residual active form of Cdt1 persists during S phase and to avoid new licensing of replicated origins, a second mechanism involving the physical interaction of Geminin with Cdt1 inhibits its activity [99]. Indeed*,* immunodepletion of Geminin from metaphase extracts permitted them to assemble licensed replication origins, suggesting that inhibition of Cdt1 by Geminin is critical for preventing origin assembly at later stages of the cell cycle [113]. Interestingly, Geminin furthermore has a positive effect on the pre-RC formation, as Geminin protects Cdt1 from ubiquitin-mediated proteasomal degradation during late G2 phase and mitosis, thereby allowing the accumulation of Cdt1 in an inactive form for the next pre-RC assembly in late M/G1 phase [110]. Geminin stabilization at late G2 or mitosis ensures that Cdt1, although at higher levels, does not re-activate the DNA replication origins [105]. In conclusion, the Cdt1–Geminin interaction, together with the regulation of Cdt1 and Geminin protein levels, is one of the main molecular mechanisms by which origin licensing is repressed during the S, G2, and M phases [110,113].

## 6. Pathological Consequences of Dysregulation of DNA Replication

Importantly, dysregulation of DNA replication licensing is linked to replication stress, one of the emerging causes of genomic instability in sporadic cancers [114]. Many oncogenes trigger replication stress by affecting the assembly of pre-RC, origin licensing, and firing, which cause replication-related errors and genomic lesions. The identification of a multitude of mutations in genes encoding replication proteins in human disease underscores the importance of faithful replication.

Autosomal recessive mutations causing loss of function of RecQ4, have been shown to cause Rothmund–Thompson syndrome (RTS) [115], a genetic disorder characterized by reduced growth and cancer predisposition.

Hypomorphic mutations in genes encoding Cdt1, Cdc6, Orc1, Orc4, or Orc6, proteins of the pre-replicative complex, are found in patients with Meier–Gorlin syndrome (MGS), characterized by primordial dwarfism, microcephaly and developmental abnormalities in the ear and patella [116,117]. Orc1 mutations were shown to affect the inhibition of cyclin E-Cdk2 kinase activity and cause centrosome reduplication or disrupt the BAH domain which affects Orc1 recruitment to the chromatin and subsequent origin licensing [118,119]. Mutations in the C-terminus of Orc6 reduce its affinity for the core ORC complex, affecting pre-RC assembly and origin licensing [120]. Although missense mutations in *ORC4* have also been identified, how these affect the pre-replicative complex is not clear yet [121,122]. For Cdt1, mutations in the C-terminus, implicated in MCM helicase complex binding, have been reported [122]. These are presumably hypomorphic mutations rather than null, since Cdt1 is an essential gene. Finally, to date only one Meier–Gorlin syndrome patient was identified with a CDC6 missense mutation, which affects the ATP binding domain, essential for its function in DNA replication [122].

Interestingly, recent publications report mutations in the *CDC45* and *GMNN* genes, encoding proteins functionally distinct from previously identified MGS-associated genes, in patients with this syndrome. Mutations in the *CDC45* result in splicing defects that reduce the amount of functional Cdc45 protein, thereby affecting the initiation of DNA replication and cell proliferation [123]. Although Geminin is formally not part of the pre-replicative complex, it does play a critical role in regulating replication. Of particular interest with respect to this review, MGS-associated mutations in *GMNN* affect the destruction box, the motif required for Geminin degradation, leading to more stable Geminin protein and persistent inhibition of DNA replication [124].

Quite strikingly, although the MCM complex plays a critical role in origin recognition, for a long time there seemed to be no Meier–Gorlin syndrome patients with MCM mutations. In addition, the fact that *MCM4* hypomorphic mutations generate chromosome instability and increased tumour incidence in mice suggested that MCM mutation results in a different phenotype [125]. However, upon very recent sequencing of a patient, diagnosed with MGS based on clinical features, biallelic mutations in the *MCM5* gene were identified: a single base deletion causing a premature stop codon and a missense mutation within a domain critical for the helicase activity [126].

The strict cell cycle-dependent regulation of several proteins involved in DNA replication has resulted in the use of these proteins as tumour biomarkers potentially used for screening of a range of cancers. Examples are PCNA, a common marker for cell proliferation and the MCM proteins. The latter, are highly expressed during the regular cell cycle but degraded in quiescent, senescent and differentiated cells, making them useful and effective additional markers of active proliferation [127,128]. Interestingly, the absence of MCMs in differentiated cells might be regulated by a post-translational mechanism, as an Mcm2-related protein fragment, possibly a cleavage product, lacking essential domains, was found in human keratinocytes [129]. In contrast, a substantial increase in MCM expression was observed in dysplasia and malignant epithelia in several types of tissues [130]. The fact that the majority of the cells stain positive for MCM in squamous intraepithelial cervical lesions suggested that cervical smear assessment might benefit from MCM immunostaining [131]. Moreover, using detection of MCM expression was proposed for the detection of colorectal cancer in colonocytes in faeces [132]. In addition to the use of MCM expression for cancer screening, expression of MCM proteins detected by immunohistochemistry can independently predict the survival in patients with a wide variety of cancers, including breast, prostate and lung tumours [133,134,135]. MCM expression generally correlates with a clinically more aggressive phenotype and shorter disease-free survival [130,136].

As may be expected given the importance of a controlled equilibrium between Geminin and Cdt1 for correct DNA replication, dysregulation of Geminin or Cdt1 have been linked to chromosomal instability, DNA replication alterations, and aneuploidy [137]. As described earlier in this review, protein levels of Geminin are strictly regulated during the cell cycle with expression limited to the early S, G2, and M phases and Geminin staining thereby only labels actively proliferating cells [99]. In accordance to this observation, Geminin was recently identified as a novel strong, independent prognostic tumour marker, and the prognosis for recovery is inversely related to the level of Geminin expression. For example, in breast cancer, high Geminin expression was strongly predictive of poor clinical outcome [138,139]. Interestingly, a recent report also demonstrated a link between USP7, a ubiquitin hydrolase regulating Geminin stability, and breast cancer-specific survival, suggesting that USP7 might be implicated in Geminin deregulation during breast cancer progression [112].

Although only few of the described examples of abnormal regulation of replication proteins in patients are related to an aberration of ubiquitin control, they undoubtedly demonstrate that correct regulation of replication proteins is an essential component in healthy individuals. Future research will establish possible novel relationships between alterations in regulation of DNA replication by (de)ubiquitination related to human disease.
